# Energy-Efficient and Reliable Face-Routing Scheme in Wireless Networks

**DOI:** 10.3390/s21082746

**Published:** 2021-04-13

**Authors:** Hyunchong Cho, Sangdae Kim, Seungmin Oh, Euisin Lee, Sang-Ha Kim

**Affiliations:** 1 Research Institute for Computer and Information Communication, Chungbuk National University, Cheongju 28644, Korea; hccho@chungbuk.ac.kr; 2 Division of Computer Science and Engineering, Kongju National University, Cheonan 31080, Korea; sdkim.cse@gmail.com (S.K.); smoh@kongju.ac.kr (S.O.); 3 School of Information and Communications Engineering, Chungbuk National University, Cheongju 28644, Korea; 4 Department of Computer Engineering, Chungnam National University, Daejeon 34134, Korea; shkim@cnu.ac.kr

**Keywords:** wireless network, geographic routing, face-routing, energy efficiency, transmission reliability

## Abstract

Face-routing is one of the reliable recovery schemes when geographic routing fails to transmit data packets. Although studies on face-routing can overcome the failure of the data transmission, they lead to much energy consumption due to frequent data transmissions between adjacent nodes for carrying out the rule of face-routing. To avoid the frequent data transmissions, several face-routing schemes have been recently proposed to transmit data packets to the farthest-neighbor node. However, they happen with many data retransmissions because the farthest-neighbor node has a relatively low transmission success ratio. To solve this problem, we propose a new face-routing scheme that determines the most appropriate neighbor node to balance the trade-off between energy efficiency and transmission reliability with two viewpoints. The first viewpoint focuses on how to increase the distance progress of the data delivery in one-hop range to enhance energy efficiency. After that, the second viewpoint focuses on how to increase the success ratio of the data delivery to enhance the transmission reliability. As a result of the simulation, it was confirmed that the proposed method achieves better performance in terms of energy efficiency than existing face-routing research, and it is better than recent face-routing research in terms of reliability and retransmission.

## 1. Introduction

In wireless networks, many researchers have paid attention to geographic routing, which is one of the efficient routing methods because it does not use the topology information of the entire network but it only uses the location information of the neighbors [1,2,3,4,5,6]. Geographic routing consists of two routing modes; one is a greedy mode and the other is a recovery mode called perimeter mode. In greedy mode of geographic routing, a node transmits a data packet to a neighbor node close to the destination in the range of a node. Since the greedy mode does not use the topology information, it uses the neighbor node information close to the destination node. When the geographic routing fails to transmit the data packet to the destination node, it changes a perimeter mode to solve the routing failure. For example, greedy perimeter stateless routing (GPSR) [6] is well known as one of the geographic routing protocols and it has two routing modes; one is greedy forwarding, i.e., the greedy mode of the geographic routing, and the other is a face-routing, i.e., the recovery mode of the geographic routing. When a node exploits GPSR, it tries to transmit data packets to the destination node by using greedy forwarding. When the node is faced with the situation where no candidate node exists for greedy forwarding, it changes to the routing recovery mode called the perimeter mode to transmit the data packet to the destination [4,5,6,7,8,9,10,11,12,13]. Face-routing is one of the perimeter methods to recover the failure of greedy forwarding [14,15,16,17]. Unlike greedy forwarding, face-routing finds the candidate nodes counterclockwise/clockwise for the generated faces based on the local communication graph. Just like finding a way when moving in one direction according to the left-handed/right-handed rule in a maze, face-routing transmits data packets to candidate nodes searched in a predetermined search direction to deliver the destination node. Since full-connected graphs can cause the routing problem, face-routing uses a local communication graph called a planar graph that consists of adjacent and shortest links with neighbor nodes in one-hop range. Unlike other routing, face-routing should transmit data packets based on planar graphs.

**Figure 1:**
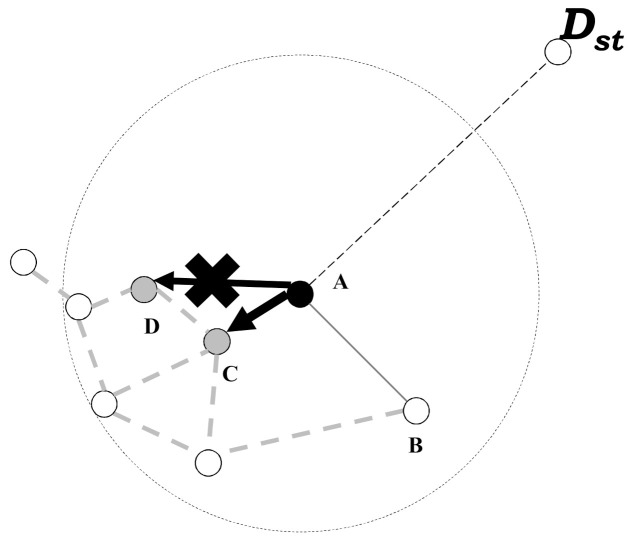
Face-routing limitation due to the generated local planar graph.

However, since face-routing uses the planar graph, it frequently results in unnecessary packet transmission in one-hop range [18,19]. As mentioned above, the planar graph consists of the shortest and adjacent links with neighbor nodes in one-hop range to avoid the routing problem. Therefore, when a node exploits face-routing, it usually follows the shortest and adjacent paths of the planar graph. Although each node can transmit a data packet to the other neighbor nodes except the shortest ones, it should determine to send the data packet to the shortest neighbors to keep the rule of face-routing. For example, as shown in Figure 1, the node A has the neighbor nodes B, C, and D in one-hop range. When the node A starts face-routing, it generates the planar graph among their neighbors. Since this planar graph consists of adjacent neighbors, A connects with the nodes B and C. In this case, according to a rule of face-routing, A cannot transmit the data packet to node D because its planar graph consist of B and C, i.e., when a node uses face-routing, even if there are farther neighbor nodes in one-hop range, it can transmit data packet to only adjacent nodes due to a rule of face-routing. This situation causes many data transmissions between candidate nodes and leads to energy depletion of the nodes in the whole network.

To solve this problem, recent studies on face-routing have proposed to reduce the energy waste of nodes by searching for the farthest-neighbor node and transmitting data packets to the searched neighbor node [18,19]. Transfer-efficient face-routing (TEF) [18], one of the recent studies finds the farthest-neighbor node by collecting the information of neighbors in the two-hop range and then generating the planar graphs. Since each node generates its planar graph and transmits its information to the source node that starts face-routing, the source node can find the farthest-neighbor node in its one-hop range. Thus, TEF can reduce the node energy consumption by transmitting a data packet to the farthest candidate node in one-hop range. In addition, energy-efficient lookahead face-routing (EELFR) [19], another of recent studies finds the farthest-neighbor node in one-hop range by using a search message at first. This scheme searches the candidate node of face-routing based on planar graphs and then, it transmits the search message instead of the data message. When the neighbor node receives the search message, it searches the candidate node of face-routing based on itself and it transmits the reply message to the starting node that starts face-routing to announce the search progress. After that, EELFR can reduce unnecessary data transmission by transmitting the data packet to the farthest-neighbor node and as a result, they can reduce the energy consumption of nodes.

However, although TEF and EELFR [18,19] can reduce the number of data transmissions by transmitting the farthest-neighbor node, they may not be efficient in face-routing. To reduce overall data transmission from the source node to the destination node, all of studies search the farthest-neighbor node in one-hop range and transmit data packets to the farthest one. However, many studies [20,21,22] have mentioned that the transmission success ratio decreases as the transmission distance of data packets increases. Based on these studies, since TEF and EELFR transmit data packets to the farthest-neighbor node in one-hop range, the link to the farthest-neighbor node usually has the worst quality in one-hop range. Moreover, due to the data transmission through the bad link quality, they lead to frequent failures of data transmission and thus cause a lot of retransmission. As previously mentioned above, since energy efficiency is inversely proportional to transmission reliability, TEF and EELFR can reduce the whole data transmission by selecting the farthest-neighbor node in one-hop range but they increase retransmission between candidate nodes of face-routing due to their node selection methods. For this reason, TEF and EELFR leads to the energy depletion of sensor nodes because they cannot reduce retransmission between candidate nodes of face-routing.

Thus, we propose an energy-efficient and reliable face-routing scheme that transmits data packets to the most appropriate neighbor node to balance the trade-off between energy efficiency and transmission reliability in face-routing. To choose the most appropriate neighbor node, the proposed scheme suggests the distance progress and the success ratio of the data delivery as two viewpoints for energy efficiency and transmission reliability, respectively. In the proposed scheme, the distance progress is calculated as the distance from the sender node to the most appropriate neighbor node, and the success ratio is measured as the link quality between the sender node and the most appropriate neighbor node. Then, the proposed scheme determines the most appropriate neighbor node to balance the trade-off by using the two suggested viewpoints different from the existing face-routing schemes.

The first viewpoint focuses on how to increase the distance progress of the data delivery in one-hop range of the sender node in face-routing to enhance the energy efficiency of nodes. In the first viewpoint, the proposed scheme tries to find the farther neighbor nodes in one-hop range to increase the distance of the data transmission more. By using a new message that has the small data size, the proposed scheme can efficiently search the farther candidate nodes of face-routing within one-hop range. By the first viewpoint, the proposed scheme can reduce the amount of entire data transmission by selecting farther neighbor nodes in one-hop range of face-routing.

After achieving the first viewpoint, the second viewpoint focuses on how to increase the success ratio of the data delivery to enhance the transmission reliability between candidate nodes in face-routing. As the transmission distance increases, the success ratio of the data transmission decreases. To address this issue, the proposed scheme selects the most appropriate node with the best link quality among candidate nodes in the first viewpoint. By the second viewpoint, the proposed scheme can reduce a lot of retransmission between the candidate nodes. As a result, the proposed scheme can improve the energy efficiency of nodes by using the first viewpoint that reduces the amount of whole data transmission from the source node to the destination node, and it can improve the transmission reliability of nodes by using the second viewpoint that reduces the amount of data retransmission between the candidate nodes in face-routing.

Through simulations conducted in given simulation components, we compare the proposed scheme with two face-routing schemes. One is GPSR, which is well known as one of the traditional face-routing schemes, and the other one is TEF, which is one of the farthest-neighbor node selection schemes in face-routing. The simulation results show that the proposed scheme has better performance than GPSR and TEF in terms of energy efficiency by increasing the transmission distance of face-routing, and it has better transmission reliability when it compares with TEF by decreasing the amount of data retransmission and the delay between nodes by selecting neighbor nodes with better link qualities.

The remainder of this paper is organized as follows. Section 2 explains the related works of face-routing regarding their history and limitations. Section 3 describes the proposed scheme to solve the limitations of previous face-routing schemes. Section 4 evaluates the performance of the proposed scheme by comparing with GPSR and TEF through simulation results. At last, Section 5 concludes the paper.

## 2. Related Work

The geographic routing is called the location-based routing and this scheme transmits the data packet to the destination node based on the location information such as GPS, self-location instead of the network topology. This routing greatly improves the efficiency of the data transmission for the destination node and it exploits the various fields such as Mobile Ad Hoc Networks (MANETs), Vehicular Ad Hoc Networks (VANETs), and Wireless Sensor Networks (WSNs) [1,2,3,4,5,6,10,13]. The geographic routing consists of two modes; one is the greedy mode called greedy forwarding and the other is the recovery mode called perimeter mode.

### 2.1. Geographic Routing

**Figure 2:**
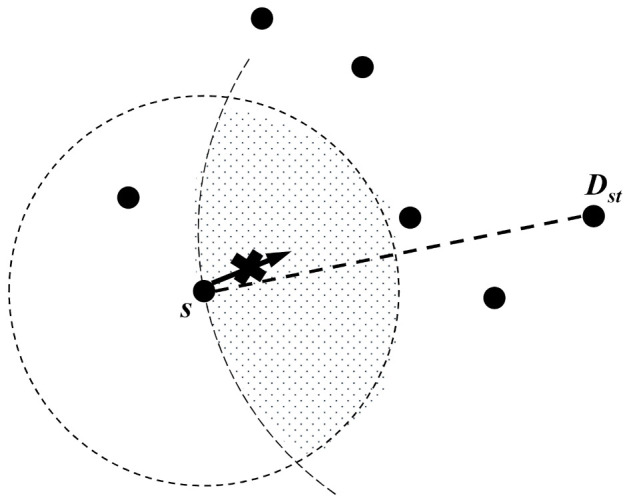
Greedy forwarding failure during the data transmission.

Greedy mode, called greedy forwarding, is a scheme that can select the closest neighbor nodes to the destination in its one-hop range. As the previous studies of face-routing assumed a radio range shape as an ideal circle surface and dealt with each method [7,8,9,10,11,12,13,18,19], we also proceed based on their studies that assume a radio range as an ideal circle shape. After the current node calculates a circle based on the virtual line from this node to the destination node, it finds the closest candidate node to the destination node in the intersection area between this circle and its radio range. Since greedy forwarding uses the local information in one-hop range, it does not need to use the entire topology information in the network. However, greedy forwarding does not always transmit data packets to the destination node due to the following limitation [6,7,10,13]. Like Figure 2, the source node ***s*** fails to transmit the data packet because there are no candidate nodes in the intersection area. Since WSN randomly places sensor nodes due to its characteristic, it frequently happens in this situation such as Figure 2. In this case, geographic routing copes with the data packet transmission failure by using perimeter mode, which is recovery mode.

### 2.2. Face-Routing

There are various perimeter modes in geographic routing and, among them, face-routing is one of the well-known perimeter modes. Unlike greedy forwarding, face-routing searches for candidate nodes according to the determined searching direction by using a local graph generated within one hop, it can greatly improve a reliability of data transmission in geographic routing [7,8,9,10,11,12,13,18,19].

To avoid transmission problems such as a routing loop state, face-routing exploits a local graph called planar graph. Planar graph is a local graph that consists of adjacent-neighbor nodes in one-hop range. Due to the characteristic of planar graphs, even if it generates locally, it has the same local graph in the corresponding region when it generates the entire planar graph in the network. Moreover, to prevent routing problems such as routing loop state, a planar graph consists of adjacent-neighbor nodes in one-hop range through the algorithm by removing connections that can cause the problem. There are three well-known methods for generating the local planar graph such as Gabriel graph (GG), relative neighborhood graph (RNG), and localized Delaunay triangulation (LDT) [16].

### 2.3. Face-Routing Research

There are several studies aimed at recovering the data transmission failure in face routing [6,14,15,16,17,18,19]. These face-routing studies can be divided into two viewpoints; one is the nearest-node selection and the other is the farthest-node selection.

The first viewpoint is to immediately transmit data packets when face-routing searches candidate nodes in the previous face-routing studies [6,14,15,16,17]. For example, GPSR [6] switches to face-routing to transmit the data packet when greedy forwarding fails. In GPSR, face-routing searches candidate nodes based on a planar graph by using the left- or right-hand rules and then, it transmits data packets to the searched candidate nodes. Greedy other adaptive face-routing (GOAFR) [15,16], one of first viewpoint examples, is a method of fully exploring the faces that intersect the line from source node to destination node, and then it searches first node found in face close to destination node. After this scheme finds the searched node, it restarts face-routing to this corresponding node. By using these face-routing studies, a source node can transmit data packet to destination node in unstable situation. However, GPSR and GOAFR have the same problem when they transmit data packets to a destination node. Since they should transmit a data packet based on a planar graph that consists of adjacent-neighbor nodes, many data transmission occurs until the packet arrives at the destination node. Moreover, since a planar graph consists of adjacent-neighbor nodes with short distances, when face-routing selects a candidate node based on planar graph, it causes many data transmissions. For this reason, due to lots of the data transmission based on the planar graph, these studies of first viewpoint have the limitation of decreasing node energy efficiency and are inefficient to a sensor node that has the limited resources [2,23].

To solve this limitation of the first viewpoint, face-routing studies related to the second viewpoint appear [18,19]. To compensate for this limitation of frequent data transmission, second viewpoint targets to the farthest-node selection. For example, TEF [18] uses local planar graphs of all neighbor nodes in one-hop range to reduce data transmission of face-routing. This scheme searches the farthest candidate node in one-hop range by receiving local planar graphs from its neighbor nodes and then, it transmits data packets to a searched candidate node. As a result, TEF improves energy efficiency than previous face-routing research by reducing data transmission of face-routing. EELFR [19], one of the second viewpoint examples, can decrease data transmission because it transmits data packet the farthest candidate node of face-routing in one-hop range. Since this scheme finds the farthest candidate nodes based on planar graphs by using the search message, it can find the farthest candidate node of face-routing in one-hop range without violation of face-routing rule. For this reason, EELFR can improve energy efficiency by transmitting a data packet to the farthest-neighbor node in one-hop range, i.e., those studies of second viewpoint can improve energy efficiency by transmitting data packets to the farthest-neighbor node in one-hop range when they compare with previous research of first viewpoint.

However, although these studies of second viewpoint improve energy efficiency of nodes by reducing the amount of the data transmission of face-routing significantly, these schemes cause another problem. Since these studies of this viewpoint select the farthest-neighbor node in data transmission, the success ratio of data transmission is significantly lower. According to the research [20,21,22], the success ratio of data transmission decreases depending on distance of data transmission. For this reason, from this new viewpoint, the farthest-node selection method causes data transmission failure because it tries to transmit a data packet to the farthest candidate node in one-hop range, i.e., since these studies of second viewpoint frequently fail to transmit data packets between the candidate nodes of face-routing, they have a higher retransmission ratio than the studies of first viewpoint. As a result, studies of second viewpoint may increase energy consumption due to data retransmission between candidate nodes of face-routing. To solve this limitation, the proposed scheme finds an appropriate node with good link quality among farther candidate nodes in one-hop range to increase energy efficiency and to keep a good link quality.

## 3. Proposed Scheme

This section introduces the several components for the explanation of the proposed scheme such as overhearing function, link quality, message type, the quality determination method between neighbor nodes, the node selection method, and example. The proposed scheme shows the two types of the message type; one is the data message used in the past and the other is new type message to search the candidate nodes of face-routing. After that, this section describes the quality determination method and the node selection method to determine the data candidate nodes and it explains the example to improve the understanding of the proposed scheme.

### 3.1. Overhearing Function

The proposed scheme aims to reduce communication cost about the searching process by using a method called overhearing. Overhearing is not an additional function, but an existing technique in MAC protocol [24]. When a node uses overhearing, it can receive other packets for other neighbor nodes. Generally, when a node transmits a data packet to a neighbor node in a local region by using wireless medium, its neighbor nodes receive the packet. After that, its neighbor node identifies the destination address of the packet, and then it determines whether it receives this packet or does not. If its node address is the same as the destination address, its node receives this packet. If its node address is not the same, its node drops this packet. If there is no corresponding process in the wireless medium, it is impossible to know which message has arrived at which node, and packet transmission is impossible except for wired installation between nodes. In this process, overhearing is a function receiving all packets, not determining packet reception according to the destination address. Overhearing is widely used in opportunistic routing [25] in wireless research and it is used in routing studies based on overhearing. We consider the total transmission power control in this scheme for a transmission from node *u* to *v* is given by [26]:

(1) $$E_{uv}=E_{ev}^{\left(xmit\right)}+E_{uv}^{\left(rcv\right)}+E_{uv}^{\left(ov\right)}=E_{txelec}+\epsilon_{amp}d_{uv}^{\alpha }+N_{uv}^{\left(ov\right)}E_{rxelec},$$

where N${}_{uv}^{\left(ov\right)}$ is the number of nodes within the communicating radius of *u* when it communicates with *v* and E${}_{txelec}$ is the transmission energy required to transmit a bit of data over the distance d${}_{uv}$. The $\epsilon_{amp}$ is the characteristic constant of the amplifier in the transmitter, and E${}_{rxelec}$ is the energy required to receive data.

The proposed scheme uses overhearing to reduce energy consumption in the searching process of candidate nodes of face-routing. Unlike EELFR [19], which transmits an additional response message every time when it finds a candidate node in the search process, the proposed scheme receives a search message by using an overhearing function and identifies a candidate node without a response message. In the proposed scheme, when a node starts face-routing, this node activates an overhearing function to receive a search message. When this node receives a search message that is out of one-hop, it stops the overhearing function and then, it tries to find the final candidate node of face-routing by using link quality.

### 3.2. Link Quality

In the proposed scheme, link quality means packet transmission success rate for each node when nodes exchange data packets themselves. There are several link quality measurement methods such as RSSI measurement, LQI measurement, and an SNR measurement. According to the WSN survey [27], since LQI and SNR measurement methods may not be accurate, the proposed scheme uses RSSI measurement method. Since the RSSI measurement method measures based on the strength of the signal and does not require additional calculations, this is suitable for sensor nodes with limited resources. In the WSN environment, sensor nodes exchange beacon messages to take information between nodes [2]. At this time, each node can calculate RSSI for other neighbor nodes. According to RSSI research [21,28,29,30,31], the signal strength gradually decreases as the distance increases. In addition, according to RSSI research [32], as the RSSI signal decreases, the packet reception rate decreases as well. In addition, since a node does not calculate the link quality of all nodes in a network, but it only calculates the link quality of neighbor nodes in one-hop range, it does not process a large number of calculations. The RSSI-based link quality measurement scheme will be managed as a table of each node in terms of memory viewpoint, but the number of indexes of this table is only the number of its neighbor nodes in one-hop range.

The proposed scheme uses link quality based on RSSI measurement and then, it can find a candidate node of face-routing. By overhearing search messages, the proposed scheme identifies candidate nodes of face-routing within one-hop range and then, it checks the link quality of its candidate nodes. After that, to select the most suitable candidate node for data transmission among candidate nodes, the proposed scheme selects the node that has the highest value by multiplying transmission distance and link quality of each searched candidate node. Since two components have a great influence on data transmission, the proposed scheme selects the candidate node that has the highest result value. As mentioned earlier, according to the WSN research [2], since data transmission is the largest in terms of node energy consumption, as the transmission distance increases, the number of end-to-end transmissions decreases. Therefore, the transmission distance has a great influence on node energy consumption. In addition, as mentioned above, since link quality influences the transmission success rate for data packets, link quality also has a great influence on node energy consumption. If the highest result value is found by multiplying the two variables, the proposed method can select the most suitable node for data packet transmission.

### 3.3. Message Classification

In the proposed scheme, there are two kinds of message type; one is the data message and the other is the search message. The data message is the same as the data packet that the node transmitted the packet by using the geographic routing. Like the data packet, the data message has the purpose that it should deliver to the destination node. As mentioned in previous research [2], a data message has the energy consumption in proportion to the size of the data message and its energy consumption is determined by the application. Compared with the search message, the data message has a larger message size than the search messages, and, with this reason, the energy consumption is also larger than the search message. In the proposed scheme, when a starting node finds the proper candidate node of face-routing, it transmits the data message to the corresponding candidate node.

Like the previous face-routing research [19], the proposed scheme exploits the search message to find all candidate nodes of face-routing within one-hop range. Unlike the data message, the search message has small message size to minimize the energy consumption of transmission. With this reason, the search message only includes the location information of the starting point of face-routing and it consumes small energy in the message transmission.

When each neighbor node receives the search message from other neighbor nodes, it checks whether the starting node included in the search message is its neighbor node. If the content of the search message is not its neighbor of the node which received the search message, this node stops to find the search progress of face-routing because its neighbor is out of the radio range of the starting node of face-routing. On the other hand, if the content of the search message is included in its neighbor, it means that this node which received the search message is the neighbor of the starting node of face-routing. Then, this node makes the local planar graph based on its neighbors and it starts face-routing centered on itself to find other candidate nodes of face-routing. After this node finds the candidate node by using face-routing, it transmits the search message to the searched candidate node. Each node repeats the search process each time it receives a search message and repeats until it finds a node that is not a neighbor of the node which received the search message.

Although the recent research of face-routing checks the farthest neighbor of a starting node by using the reply message, the proposed scheme does not use the reply message but it uses the overhearing of each node, i.e., after the starting node of face-routing transmits the search message to its neighbor node, it can know that its neighbor nodes transmit the search message to other nodes. Based on this overhearing, this starting node identifies which the neighbor node has received the search message and then it can find the candidate nodes of face-routing in one-hop range of the starting node. Moreover, this starting node can know when the search process ends by using the overhearing. Since the starting node of face-routing receives the search message through the overhearing, it can know the destination of each search message. When this starting node receives the search message and identifies that the destination of the search message is out of range, it stops the overhearing and starts the final candidate node selection of the data transmission.

If a search message is lost due to other factors such as noise during transmission, a node that performs the overhearing function may not receive this search message. Since this node continues to wait until it receives a search message that has a destination outside one-hop radio range, it waits a certain amount of time. If this does not receive a search message after a certain time, it determines that a search message loss has occurred. After that, this node searches for a recently registered candidate node in a candidate node list generated by receiving search messages from its neighbor nodes. Since this is the time for this registered node to transmit a search message to a next candidate node of face-routing, when a problem such as message loss occurs, a node that performs the overhearing function transmit a search message to this node to restart a searching process of face-routing. Thereafter, a node that performs the overhearing function continues to wait for a search message, and a node that receives a search message searches for a candidate node of face-routing.

### 3.4. Quality Calculation between Candidate Nodes

After the starting node of face-routing finds all candidate nodes in its radio range by using the search message, it selects the final candidate node that will transmit the data message from the starting node. This process has the purpose that delivers the data message to the farther neighbor node with high link quality. As mentioned above, one of large energy consumption in a node is data transmission and it means that the average energy consumption of the node increases when the amount of data transmission increases, i.e., as the proposed scheme reduces the amount of the entire data transmission by transmitting the data message to a farther neighbor node, the average energy consumption of the node also decreases. Two previous studies of face-routing can reduce the average energy consumption of the node because they focus on the data transmission of face-routing to the farthest-neighbor node. For this reason, the proposed scheme selects a farther neighbor node to reduce the average energy consumption of the node in data transmission.

Moreover, the proposed scheme considers the link quality when it selects the candidate node of data transmission. Since the selected farthest candidate node may not be suitable for the data transmission of face-routing, the proposed scheme considers the link quality between the neighbor nodes additionally. If the starting node transmits the data packet to the farthest-neighbor node with low link quality, it may fail to send the data message to this neighbor node. For this reason, in the proposed scheme, the starting node of face-routing identifies the link qualities of its neighbors and uses these qualities information to select the candidate node about the data transmission. After that, this starting node identifies the candidate nodes through face-routing by using the search message and it uses the link quality of selected candidate nodes to select the final candidate node for data transmission.

After that, the proposed scheme selects a final candidate node of the data transmission by using the best result that multiplies the transmission distance and the link quality of searched candidate nodes. For example, this paper assumes that the node S starts face-routing and the node A is the farthest-neighbor node. The node B is the intermediate neighbor node between the node S and the node A and the link quality of two neighbor nodes is the same. In this case, the node S can select the neighbor node A as the final candidate node of data transmission because the node A has better result than the node B. Moreover, this paper assumes that the distance of the node A and the node B is same and the link quality of the node A is 90% and the link quality of the node B is 100%. In this case, the node S can select the neighbor node B as the final candidate node of data transmission because the node B has the better result than the node A. For this reason, the proposed scheme can finally select the candidate node for the data transmission by using the result that multiples the distance and the link quality of each neighbor node.

### 3.5. Node Selection Algorithm for the Energy-Efficient Face-Routing

The proposed scheme exploits the Algorithm 1 to search the candidate node for the transmission of the data message from the starting node of face-routing. The source node S means the node that fails the data transmission by using greedy forwarding, the node **v** means the node searched by face-routing, the group **V** is the group consisted of the searched node **v**, the value of *v_result* is the quality result value of each neighbor node, the value of *max_result* is the maximum value of the quality result value among the neighbor nodes, and the value of *max_i* is the index of the *max_result* value. In the proposed method, if the *wait* value is set to 1, the starting node S has a switch that waits for a search process for the candidate node of face-routing.

Algorithm 1 performs when a starting node S starts a face-routing, one of the perimeter modes in geographic routing. In this case, a node S generates a local planar graph based on its neighbor nodes. After that, a node S tries to search a candidate node of face-routing based on this generated planar graph. As shown in the Algorithm 1, *make_planar_graph()* means that starts to generate the local planar graph, and *search_candidate()* means that tries to search the candidate node based on the generated local planar graph. If a node S cannot find a candidate node of face-routing in this process, it means that this node is an isolated situation in the network, therefore, it announces routing failure to a node that receives a data message. This paper does not deal with this case since it focuses on a new face-routing scheme. When a node S finds a candidate node, it transmits a search message to the discovered candidate node. After that, to listen to the search process by the overhearing function, proposed scheme sets the *wait* value to 1 and it initializes that the value of *max_result* sets to 0. Until *wait* value sets to 0, a node S continues to listen to a search message with the overhearing function. By using the overhearing function, a node S can know the discovered candidate nodes of face-routing by receiving the search message. In addition, if a node S receives the search message that destination is not its neighbor, it means that it is time to stop the search progress because this search message will be transmitted out of range of a node S. In this case, node S checks the candidate nodes included in the group **V** and selects a final candidate node among these candidate nodes for the data transmission.
**Algorithm 1: **Node selection algorithm
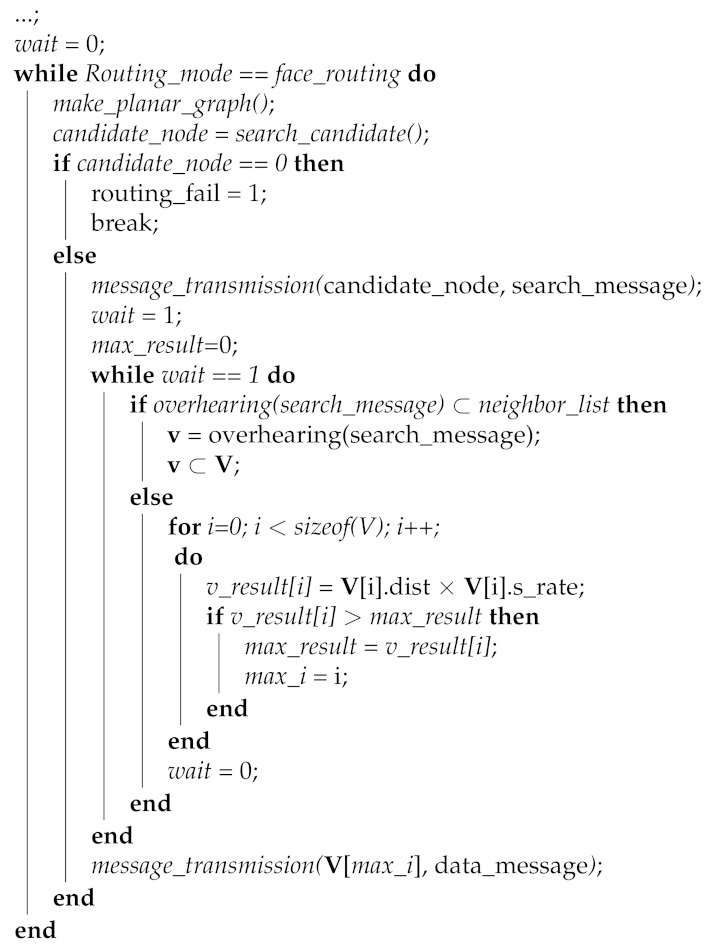


According to an Algorithm 1, the scheme shows how to select a final candidate node for the data transmission as follows. The proposed scheme sets the value of *v_result* for each candidate node, multiplies the value by distance and link quality of each candidate node, and stores the result value. This is because the two variables, the transmission distance and the link quality of each candidate node, have a great influence on the data message transmission of face-routing in the proposed scheme. If a node transmits a data message to the farther neighbors, it can reduce the total amount of data transmission from a source to a destination node. In addition, the higher the candidate node has link quality, the lower the proposed scheme has the probability of retransmission. For this reason, the proposed scheme selects a candidate node that has the highest result by multiplying two variables and, thus, it can increase the efficiency of data transmission. Therefore, the proposed scheme sets the *v_result* value for each node from the first candidate node to the last candidate node in a node group **V**. In addition, if one of the *v_result* values is greater than the maximum value of *max_result*, the proposed scheme updates it with the maximum value to find the maximum value of *v_result*. When the proposed scheme finishes this process and it identifies the maximum value of *max_result*, the *wait* value sets to 0 to stop the overhearing function. After that, it delivers a data message to a final candidate node that has the highest value of the *max_result*.

Moreover, when a neighbor node receives a search message, the proposed scheme follows Algorithm 2. Like Algorithm 2, when a node receives the search message from its neighbor nodes, it checks the information included in a search message. As mentioned above, a search message has the information of the initiated node of face-routing. Algorithm 2 identifies the node that has content of the search message by using the function called *check(search_message)* and it sets this information to **v** value. After that, a received node checks whether the node information, which is the content of the search message, is included in the neighbor node list. In Algorithm 2, the received node checks that this information is its neighbor by using the function called *match_neighbor*(**v**, neighbor_node_list) and this result sets to the *v_result* value. If this node information is not a neighbor of the current node, a received node stops the searching process for candidate nodes of face-routing. Since this situation is out of a radio range of the node initiating face-routing, and the searching process is stopped because no more candidate nodes are found. Therefore, if the *v_result* value is 0, it means that **v** is not a neighbor node and it is time to stop this searching process of face-routing. In this case, the proposed scheme uses the command of the break to stop the searching process of face-routing.
**Algorithm 2: **Received neighbor operation algorithm
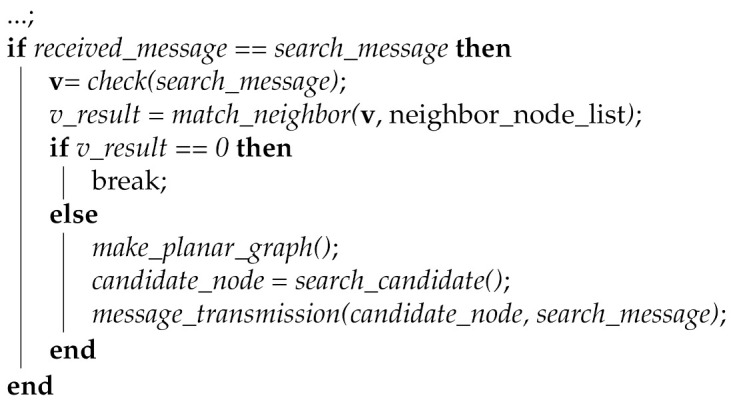


On the other hand, if this node information is included in the neighbor node list, a received node generates the local planar graph centered on itself. In Algorithm 2, if the *v_result* value is not 0, it means that **v** is the neighbor of the current node. Algorithm 2 uses the function of *make_planar_graph()* to generate a local planar graph centered on the current node. After the current node generates a local planar graph, by using the function of *search_candidate()*, it searches a candidate node of face-routing based on the generated planar graph. After Algorithm 2 searches for the candidate node of face-routing through the function of *search_candidate()*, it sets the searched candidate node to the value of *candidate_node*. Then, to transmit the search message to a discovered candidate node, Algorithm 2 uses the function of *message_transmission()* and sends a search message to the discovered candidate node by using face-routing.

### 3.6. Example in the Proposed Face-Routing Scheme

**Figure 3:**
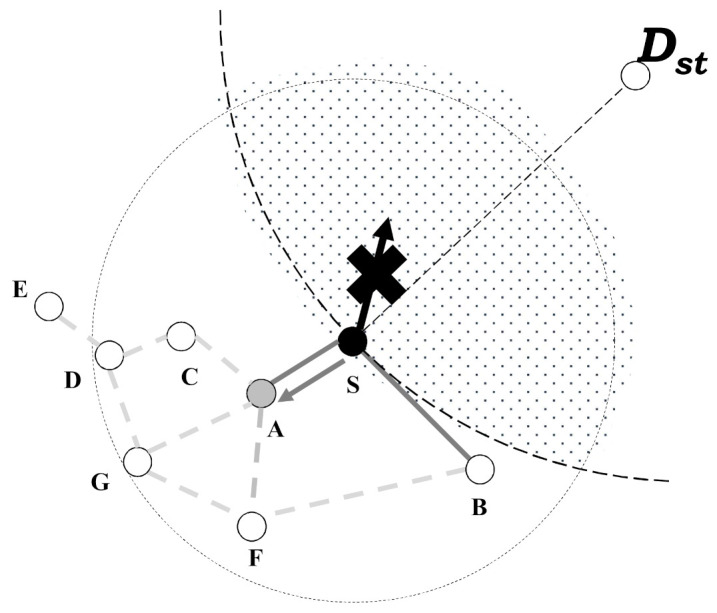
After the node S fails greedy forwarding, it finds the candidate node A by using face-routing with the search message.

The example by using the proposed scheme is as follows. Like Figure 3, the node S tries to transmit the data message to $D_{st}$ by using greedy forwarding in the geographic routing but it cannot find its candidate node of greedy forwarding. Therefore, the node S fails the data transmission to $D_{st}$ and it changes the routing mode to face-routing, i.e., the recovery mode of the geographic routing, to transmit the data message to the $D_{st}$.

As mentioned in Algorithm 1, the starting node S of face-routing generates the local planar graph centered on itself. In WSN, each node exchanges the information between its neighbor nodes by using the beacon message and then the proposed scheme uses this process to generate the local planar graph based on this exchanging information between its neighbor nodes. After that, the starting node S attempts face-routing about the destination node $D_{st}$ based on the generated planar graph to find the candidate nodes of face-routing. In this example, face-routing searches the candidate nodes of face-routing in the counterclockwise direction. Then, the starting node S finds the candidate node A by using face-routing and it includes this candidate node A to the candidate node list of the data transmission as Algorithm 1 includes the searched candidate node of face-routing to the group **V**.

After a node S transmits the search message to a candidate node A, it changes the wait state to receive a search message by using the overhearing function. Until a node S finds a last node of face-routing in the one-hop range, it continues to receive the search message. Then, as mentioned Algorithm 2, a node A receives a search message from a node S and it checks the information of a search message to identify an initiated node of face-routing. In this case, a node A should check whether it is the neighbor of a node S or not. As shown in Figure 3, since a node A is in a radio range of a node S, node A identifies that node S is a neighbor in this process. After that, a node A tries face-routing centered on itself to find a next candidate node of face-routing.

**Figure 4:**
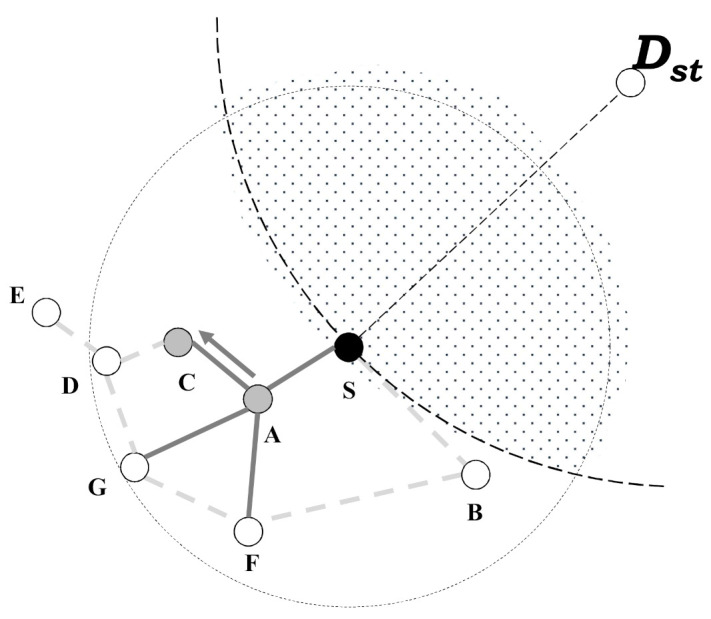
While the node A finds the candidate node C during face-routing, the node S identifies the node C by using the overhearing of the search message.

In Figure 4, since a node A is a neighbor of a node S, it starts to generate its local planar graph based on its neighbor nodes. After a node A generates a local planar graph, it starts to search a candidate node based on the generated local planar graph by using face-routing. As shown in Figure 4, a node A finds a candidate node C and it transmits a search message to a node C to find a next candidate node of face-routing. At this time, a node S can receive a search message from a node A by using the overhearing function, i.e., through this process with the overhearing function, a node S can figure out which node transmits a search message. After that, a node S can identify whether node C is its neighbor. When a node S checks its neighbor node list, it can find a neighbor node C. Therefore, a node S includes a node C in a candidate node list of data transmission. Like a case of node A, a node C generates its planar graph based on its neighbor nodes. Then, a node C finds a candidate node D by using face-routing, and it transmits a search message to a node D. In this case, a node S receives this search message through the overhearing function and it identifies whether a node D is a neighbor or not. As a result of identification, since a node D is a neighbor of a node S, a node S includes in D to a candidate node list for data transmission and continues to wait for the search messages.

**Figure 5:**
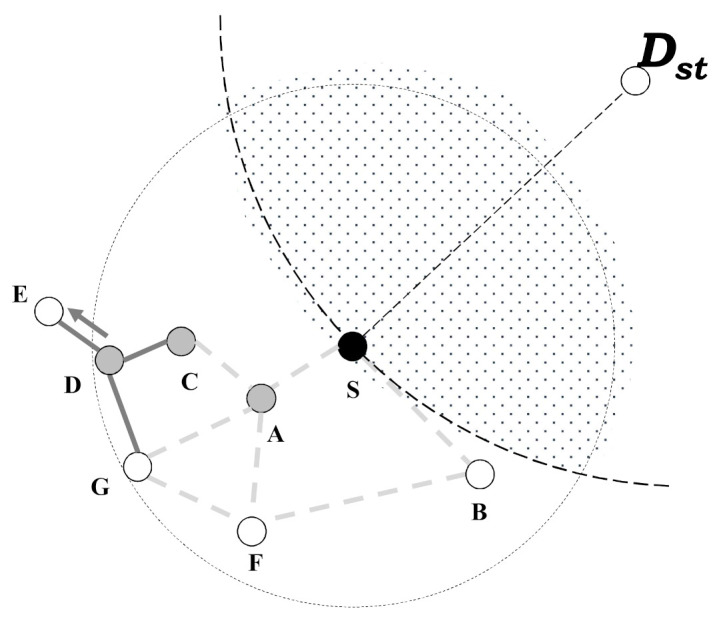
In this search progress, the node S can identify that the node E is not its neighbor node.

As the following Figure 5, when a node D receives a search message from a node C, it checks the contents of a search message. Among the contents of a search message, a node D finds a node information that initiates a search message and checks whether it is included in its neighbors. Since a node D is included in one-hop range of a node S, it starts to generate a local planar graph based on its neighbor to find the next candidate node of face-routing. After that, a node D finds a candidate node E and it transmits a search message to a node E. At this time, a node S receives this search message by using the overhearing function and then it identifies a destination of a search message. In this case, a node S knows that a destination of a search message is a node E and then, it identifies whether a node E is a neighbor of a node S or not. Like Figure 5, since a node E is not a neighbor of a node S, a node S stops the overhearing function and it checks a candidate node list of data transmission. This list of data transmission consists of the node A, node C, and node D. When a node E receives a search message from a node D, it also checks the content of the search message to find information about the initiated node. As shown in Figure 5, since a node E is not included in one-hop range of a node S, it finds that a node S is not its neighbor. It means that it is unnecessary to perform a search process no more because it is out of radio range of a node S. For this reason, the node E stops to perform the search progress.

After that, a node S tries to find an appropriate candidate node for data transmission among the identified candidate nodes, which are A, C, and D. To determine a final selection of data transmission, a node S identifies the transmission distance and the link quality of each candidate node according to an Algorithm 1. Those variables have already been identified when a node S exchanges its beacon message to its neighbor nodes to know the information of its neighbors. For example, it is assumed that a distance from a node S to a node A is 3m, a distance of a node C is 7m, and a distance of a node D is 9m. In addition, it is assumed that the transmission success rate of a node A from a node S is 100%, a case of a node C is 100%, and a case of a node D is 70%. As following an Algorithm 1, the proposed scheme can calculate a result value of each node. Then, a node A has a result value of 3, a node D has a result value of 7, and a node E has a result value of 6.3. Comparing the result of each node, a node D has the highest result value among three candidate nodes. Therefore, a node S transmits a data message to a node D.

Although a node S can transmit a data message to the farthest-node D to reduce the amount of data transmission, the proposed scheme selects to transmit a data message to node C to reduce data retransmission. The lower the link quality, the higher the data transmission failure rate. Therefore, when a node has low link quality, it may require additional data retransmission. To avoid this situation, the proposed scheme selects a node that has the higher link quality.

## 4. Performance Evaluation

This section compares the performance of each face-routing scheme through the simulation results. It compares the performance of three schemes; one is the GPSR [6] that represents one of the traditional face-routing studies, another is TEF [18], which represents one of the farthest candidate node selection studies, the other is the proposed scheme. This section describes the required simulation environment at first, and then it calculates the simulation results based on its environment, and it compares the calculated performance with each face-routing scheme.

### 4.1. Simulation Environment

This section describes the simulation environment to explain the performance evaluation in detail. This paper exploits the MATLAB simulator [33] to compare the existing face-routing studies. There are several simulation parameters described in Table 1 to use in the section. Since this simulation requires only face-routing part in the geographic routing, greedy forwarding of the geographic routing is omitted and only face-routing method is applied to the simulation. Each node initially exchanges its location information to each neighbor node with IEEE 802.15.4 physical and MAC layers.

**Figure 6:**
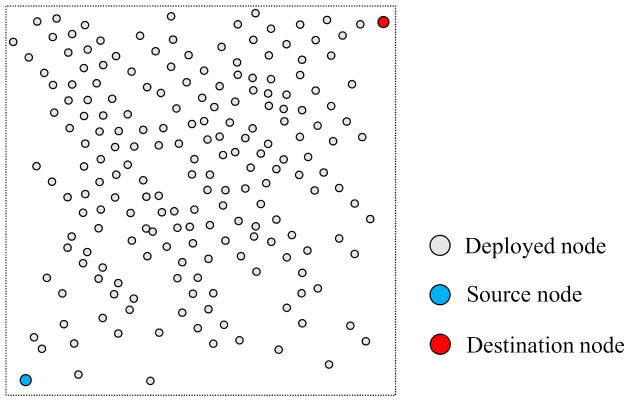
Example among random deployment of nodes in simulation.

The nodes were placed in a square area of 80 × 80 m${}^{2}$ area and randomly arranged according to the characteristics of the WSN like the Figure 6. When the simulation performs according to the change in the number of nodes, it performs to change from 200 nodes to 500 nodes. The change in the number of nodes means the node density in the network and then, it is possible to grasp the effect of each face-routing research on the dense environment of the nodes.

In addition, the end-to-end distance, which is the transmission distance from the source node to the destination node, sets to 70 m. When the simulation performs according to the change in the end-to-end distance, it performs to change from 60 m to 74 m. The change according to the end-to-end distance means the change in the overall data transmission distance from the source to the destination node and then, as the distance increases, the number of selected candidate nodes of face-routing increases. Therefore, this simulation can grasp the effect of the increased number of face-routing candidate nodes.

The one-hop radio range of the node sets to 30 m, and when the simulation performs according to the change in the radio range of the node, it performs to change from 30 m to 40 m. The change according to the radio range of the node means an increase in the number of the candidate nodes of face-routing within one-hop range. Accordingly, it is possible to grasp the influence of the increase in the number of candidate nodes of face-routing within one-hop as the radio range increases. This simulation has no significant effect on existing face-routing studies of the nearest-node selection like the GPSR [6], but it has a great effect on face-routing research of the farther node selection such as TEF [18] and the proposed scheme.

In this simulation, we compare three studies of face-routing. One is GPSR [6] and is one of the traditional face-routing techniques in WSN. Also, since GPSR is one of the nearest-node selection methods in face-routing research, it is selected as a simulation comparison target. Another is TEF [18], which is one of the techniques that improved traditional face-routing research along with GPSR. TEF is one of the farthest-node selection methods in face-routing research. Since this paper is the main target paper to be solved with the proposed scheme, it was selected as a simulation comparison target. The last one is the proposed method, and, therefore, the performance evaluation is conducted with those three simulation targets.

There are three variables used in each simulation: one is the number of nodes, another is end-to-end distance between source node and destination node, and the other is the radio range. The first simulation variable is the number of nodes deployed in the network. As the number of nodes increases, the density of a region increases as well, so that each simulation result value can be compared and analyzed according to this change. The second simulation variable is an end-to-end distance from a source node to a destination node. This distance means a geographic distance between a source node and a destination node calculated by the Euclidean distance formula. As this distance increases, the number of data transmissions increases, so that each simulation result value can be compared and analyzed according to this change. The third simulation variable is a radio range of each node that can transmit data packets. As the radio range of a node increases, the number of searched candidate nodes for face-routing increases, so that each simulation result value can be compared and analyzed according to this change.

There are three results calculated from each simulation: one is the average energy consumption, another is the average transmission success ratio, and the other is the average retransmission ratio for each node. The first simulation result is average energy consumption of the node and this is an average value of total energy consumption of data transmission for each face-routing research. When source and destination nodes are randomly selected and a data packet is transmitted, simulation measures the consumed energy consumption for each node and obtains an average value of this results. Among the communication parts related to energy consumption, some parts may be related to the physical layer or MAC layer. However, since we focus on a part related to a routing part in a network layer, the energy consumption model of the physical layer or MAC layer part was not included in the simulation. Through this process, it is possible to determine which research among the simulation targets consumes more energy and which research consumes less energy. The second simulation result value is average transmission success ratio. This value is an average value of the number of success transmission times measured when simulation transmits data packets from source to destination node. Through this process, the simulation can determine which research has a high transmission success ratio and which research has a low transmission success ratio. The third simulation result value is average retransmission ratio per node. This value is an average value of the retransmission ratio when simulation transmits data packets from source to destination node for each face-routing research. Through this process, when each node transmits data packets in simulation, it is possible to determine which research has a high retransmission ratio and which research has a low retransmission ratio. For the accuracy of simulation, this paper calculates the average results by repeating 200,000 times for each simulation condition on a graph.

### 4.2. Simulation Results

**Figure 7:**
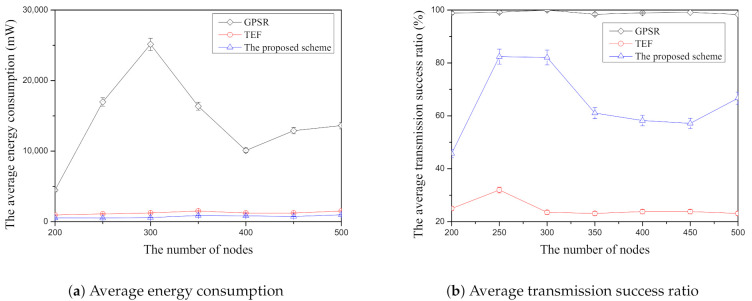
The comparison of the average energy consumption (Figure 7a) and the average transmission success ration (Figure 7b) according to the number of nodes.

Figure 7a shows the results of the average energy consumption according to the number of nodes in each face-routing research through the simulation. In Figure 7a, the GPSR, one of the nearest-node selection schemes of face-routing, is the highest average energy consumption among three face-routing techniques. Since this scheme selects the first adjacent-neighbor node when it searches a candidate node by using face-routing, its candidate node consists of the short distance of the data transmission. For this reason, GPSR usually has the largest number of data transmissions compared with the other two face-routing techniques and it results in the highest average energy consumption. Another face-routing technique is TEF [18], which is one of the farthest-node-selection techniques of face-routing and which has a lower average energy consumption than GPSR. Unlike GPSR, since TEF can search and transmit the data message to the farthest-neighbor node in one-hop range, it can reduce the amount of data transmission compared with GPSR and have a lower average energy consumption. However, TEF is larger than the proposed scheme since this scheme selects and transmits the farthest-neighbor node in one-hop range. As mentioned above, as the transmission distance increases, the data transmission success ratio decreases [20,21,22]. Since this scheme occurs the data retransmission due to the farthest-node selection, this scheme increases its average energy consumption compared with the proposed scheme. The proposed scheme has the lowest energy consumption compared with the two face-routing techniques. Since this scheme selects an appropriate farther neighbor node by considering its transmission distance and its data transmission success ratio, it can reduce the amount of data transmission and retransmission. Since all face-routing studies have the environment of random node deployment and the random selection of the source and the destination, their results have the fluctuation of the average energy consumption according to the number of nodes. To sum up, GPSR has the largest energy consumption compared with other techniques, TEF is the second largest consumption, and the proposed scheme has the lowest energy consumption in Figure 7a.

Figure 7b shows the results to present the average transmission success ratio according to the number of nodes among face-routing research. In simulation, the calculated average transmission success ratio is the represented percentage part how much get the success transmission when a candidate node receives from the total number of data message transmissions from the starting node of face-routing. This figure shows that GPSR has the highest average success ratio among three face-routing techniques because of its candidate node selection as mentioned above. Since this scheme selects adjacent candidate nodes based on the planar graph during face-routing, it has a highest average transmission success ratio. On the other hand, TEF has the lowest average transmission success ratio because it searches the farthest candidate node from a starting node of face-routing and transmits the data message to the selected farthest neighbor. As mentioned above, the data transmission success ratio decreases as it transmits the data message to the farther distance in one-hop range [20,21,22]. Therefore, since this scheme retransmit the data message to each candidate node, it has the lowest average transmission success ratio. The proposed scheme is better performance of average transmission success ratio compared with TEF because it selects the candidate nodes with high data transmission success ratio among the farther neighbor nodes in one-hop range. However, compared with GPSR, the proposed scheme is lower average transmission success ratio because it tries to select the farther neighbor nodes to reduce the data transmission. As mentioned above, due to the random node placement and random selection of the source and destination nodes, all the face-routing research has the change of the average data transmission according to the number of nodes but, overall, the average transmission success ratio of GPSR has the highest average transmission success ratio compared with other two schemes. Moreover, TEF is the lowest average transmission success ratio and the proposed scheme is located in the middle.

**Figure 8:**
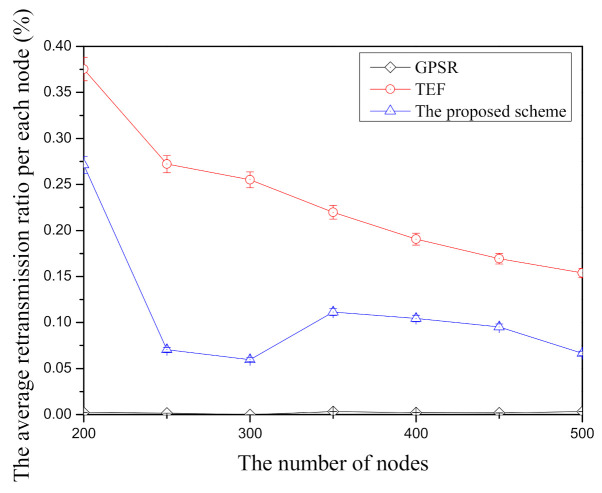
The comparison of the average retransmission ratio per node according to the number of nodes.

Figure 8 shows the result of the average retransmission ratio per node according to the number of nodes in face-routing research. In this figure, GPSR has the lowest average retransmission ratio per node among face-routing research because of its node selection scheme. Therefore, since the previous face-routing research selects the adjacent-neighbor nodes based on planar graphs to prevent from a routing problem such as the routing loop state, GPSR usually transmits the adjacent-neighbor nodes that have higher data transmission success ratio when it transmits the data message. Therefore, GPSR has the lowest retransmission ratio per node compared with other face-routing research in the simulation. On the other hand, the TEF has the highest average retransmission ratio per node among face-routing research. It is because TEF searches the farthest-neighbor node in one-hop range and transmits the data message to searched neighbor nodes to reduce the data transmission. However, TEF has the highest data message retransmission ratio due to its node selection scheme. Since the data transmission success ratio gradually decreases as the distance of data transmission is far from one-hop range, this scheme has the highest average retransmission ratio per node. The proposed scheme has the middle position between GPSR and TEF. Unlike TEF, the proposed scheme selects the farther neighbor nodes with the higher average data transmission success ratio and transmits the data messages to the selected neighbor nodes. For this reason, the proposed scheme can reduce the average retransmission ratio per node when it compares with TEF. However, since the proposed scheme selects neighboring nodes that are farther away than GPSR, the retransmission rate is higher than that of GPSR. Like other figures in the simulation, due to the random node deployment and random selection about source and destination, there are lots of changes of the average retransmission ratio per node in all face-routing research according to the number of nodes. Nevertheless, among three face-routing studies, GPSR has the highest average retransmission ratio per node compared with other face-routing techniques, TEF has the lowest average retransmission ratio, and the proposed scheme is in the middle in this Figure 8.

**Figure 9:**
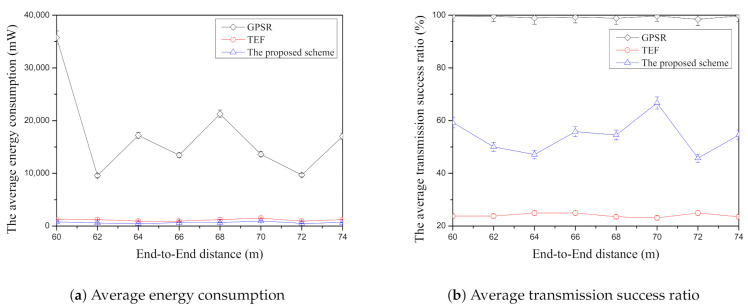
The comparison of the average energy consumption (Figure 9a) and the average transmission success ratio (Figure 9b) according to end-to-end distance.

Figure 9a shows the average energy consumption according to end-to-end distance among face-routing research. In this figure, GPSR has the highest average energy consumption compared with other face-routing research. As mentioned above, since this scheme selects the adjacent-neighbor nodes by using previous face-routing research, their nodes should transmit the data message frequently due to their short transmission distance. It means that GPSR may increase the amount of data transmission when the end-to-end distance increases and it may increase the average energy consumption. TEF has better energy efficiency when it is compared with GPSR. Since TEF transmits the data message to the farthest-neighbor node in one-hop range, it can reduce the amount of data transmission significantly. However, although TEF can reduce the amount of data transmission, it increases the data retransmission ratio because of the farthest-node selection with the lowest transmission success ratio. The proposed scheme is the lowest energy consumption compared with other face-routing research. By selecting the farther candidate nodes with the high transmission success ratio, it can reduce more data transmission than GPSR and reduce more data retransmission ratio than the TEF. As a result, the proposed scheme can decrease the average energy consumption significantly in this simulation. As mentioned above, there are random node placement and the random node selection of the source and the destination and each face-routing research has the various changes of the average energy consumption due to these environments. However, although there are different results of each face-routing research through these changes, among three face-routing techniques, GPSR has the highest average energy consumption, TEF is the second largest energy consumption, and the proposed scheme has the lowest energy consumption as shown in Figure 9a.

Figure 9b shows the average transmission success ratio according to the end-to-end distance among face-routing research. GPSR has the highest average transmission success ratio in this Figure 9b because of its node selection method. As described in the previous paragraph, since face-routing is based on the planar graph consisting of the adjacent-neighbor nodes in one-hop range, GPSR has the highest average transmission success ratio among face-routing research. TEF has the lowest average transmission success ratio in Figure 9b because this scheme searches the farthest-neighbor nodes in one-hop range and transmits the data message to them. As mentioned above, since the data transmission success ratio decreases as the transmission distance increases, TEF frequently retransmits the data message to the searched candidate nodes of face-routing. In simulation, the proposed scheme has the better average transmission success ratio when it is compared with TEF. Unlike GPSR and TEF, the proposed scheme considers two conditions; one is the distance and the other is the data transmission success ratio. Since the proposed scheme selects the farther neighbor with the high data transmission success ratio, it is better average transmission success ratio than the TEF. However, since the proposed scheme tries to select the farther neighbor nodes in one-hop range, it has a lower average transmission success ratio than GPSR as shown in Figure 9b. As mentioned previous figures in simulation, although there are random node placement and the random selection of the source and destination node, GPSR is highest average transmission success ratio, TEF is the lowest average transmission success ratio, and the proposed scheme is located in the middle between GPSR and TEF.

**Figure 10:**
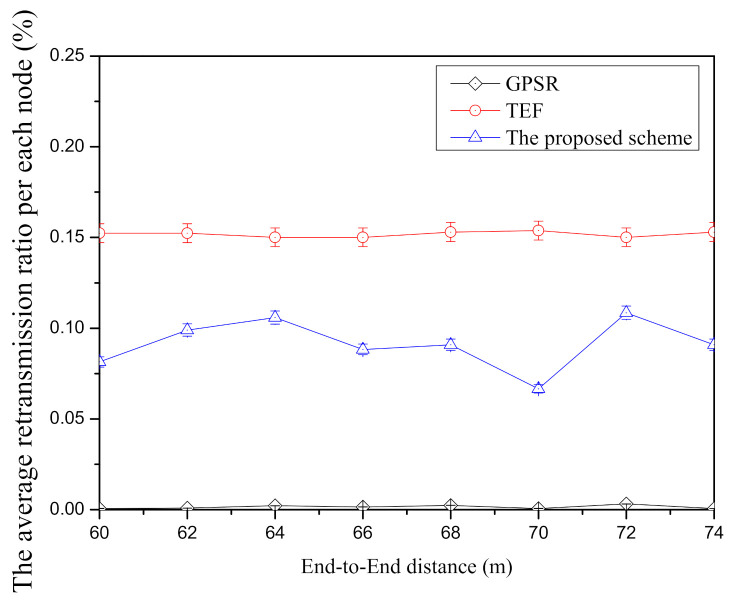
The comparison of the average retransmission ratio per node according to end-to-end distance.

Figure 10 shows the average retransmission ratio per node according to end-to-end distance in each face-routing technique. In GPSR, it is the lowest average retransmission ratio per node compared with other face-routing research since this scheme transmits the adjacent-neighbor nodes in planar graph. By using the planar graph, GPSR can avoid a routing problem such as the routing loop state and it can increase the data transmission success ratio because its transmission distance between the candidate nodes is short. On the other hand, TEF has the highest average retransmission ratio among three face-routing techniques since this scheme selects the farthest candidate nodes in one-hop range unlike GPSR. As mentioned above, when the transmission distance is far from the starting node of face-routing, it gradually decreases the transmission success ratio and, therefore, this scheme has a lot of retransmission ratio due to its node selection. Compared with TEF, the proposed scheme is lower average retransmission ratio per node because it tries to select the candidate nodes with the high data transmission success ratio to reduce the retransmission between the candidate nodes. However, since the proposed scheme selects the farther neighbor nodes to reduce the amount of data transmission from source to destination node, it has a higher average retransmission ratio per node compared with the GPSR. Although Figure 10 has the several changes such as the random node deployment and the random node selection of the source and the destination, this figure shows that the GPSR has the lowest average retransmission ratio among three techniques of face-routing, TEF has the highest average retransmission ratio, and the proposed scheme is located in the middle between two face-routing techniques.

**Figure 11:**
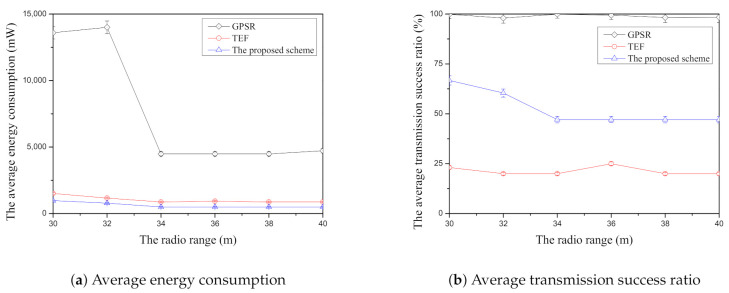
The comparison of the average energy consumption (Figure 11a) and the average transmission success ratio (Figure 11b) according to radio range.

Figure 11a shows the average energy consumption according to the radio range compared with face-routing research. Like Figure 11a, GPSR has the highest average energy consumption compared with two comparisons because this scheme is based on the planar graph when it finds the candidate nodes of face-routing. As mentioned above, the planar graph consists of the nodes that are close to each other and, therefore, this scheme usually transmits the data message to the short distance between the candidate nodes. For this reason, this scheme has a lot of data transmission due to its candidate node selection when it is compared with other face-routing research. In addition, GPSR has the highest average energy consumption regardless of the change of the radio range because the planar graph usually consists of the closest nodes in one-hop range. On the other hand, TEF can reduce the average energy consumption significantly compared with GPSR because it selects to transmit the farthest-neighbor node in one-hop range. Moreover, when the radio range increases in the simulation, TEF can reduce more average energy consumption than the GPSR because it can reduce the amount of transmission by transmitting the data message to the farthest-neighbor nodes in one-hop range. However, TEF increases the data retransmission because it usually transmits the data message to the farthest-neighbor nodes with lowest data transmission success ratio. Unlike other face-routing research, the proposed scheme can reduce the average energy consumption by selecting the farther neighbor nodes in one-hop range and it can also reduce the average energy consumption by selecting the final candidate node of the high average data transmission success ratio among them. In addition, the proposed scheme decreases the average energy consumption according to the increase of the radio range described by Figure 11a because this scheme can select more suitable neighbor nodes of face-routing in one-hop range when the radio range increases. To sum up, among face-routing research, GPSR has the highest average energy consumption, TEF has the second largest average energy consumption, and the proposed scheme has the lowest average energy consumption in simulation.

Figure 11b shows the average transmission success ratio according to the radio range in each face-routing scheme. As shown in Figure 11b, GPSR has the highest average transmission success ratio among three face-routing techniques because of the planar graph. This scheme should follow the planar graph that consists of the adjacent-neighbor nodes in one-hop range and determine the candidate nodes of face-routing with this generated graph. As mentioned above, since this scheme transmits the data message to the candidate nodes that are close to each other, it can maintain the higher average transmission success ratio. For this reason, GPSR has the better data transmission success ratio compared with other face-routing research as shown in corresponding simulation results in Figure 11b. On the other hand, the TEF has the lowest average transmission success ratio because it usually transmits the data message to the farthest candidate nodes with the low data transmission success ratio in one-hop range. Since this scheme searches the farthest-neighbor nodes in one-hop range, it frequently retransmits the data message to the selected candidate nodes because its data transmission success ratio decreases according to the farther transmission distance. The proposed scheme has the better average transmission success ratio than TEF in Figure 11b. Since the proposed scheme selects the candidate nodes that have the farther distance with the high average transmission success ratio in one-hop range, it can reduce the amount of data retransmission of face-routing. Therefore, the proposed scheme can increase the higher average transmission success ratio than the TEF. However, similar to the recent research such as TEF, since the proposed scheme selects the farther neighbor nodes in one-hop range, its average transmission success ratio is lower than GPSR. Therefore, as shown in Figure 11b, among three face-routing techniques, GPSR has the highest average transmission success ratio, TEF has the lowest average transmission success ratio, and the proposed scheme is located in the middle position between GPSR and TEF.

**Figure 12:**
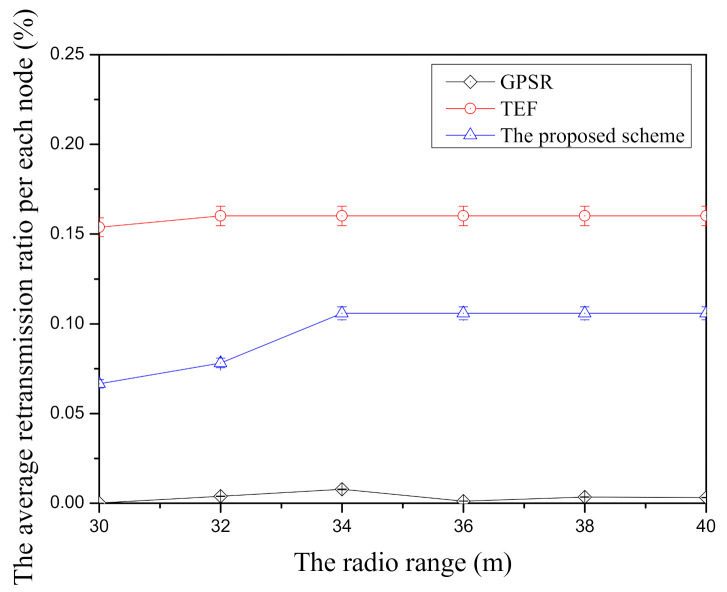
The comparison of the average retransmission ratio per node according to radio range.

Figure 12 shows the average retransmission ratio per node according to the radio range in face-routing research. In GPSR, it has the lowest average retransmission ratio per node among three face-routing technique because of its candidate node selection based on the planar graph. Since face-routing is based on the planar graph when it transmits the data message, GPSR usually selects the closest neighbor nodes with the starting node of face-routing and it has a small retransmission ratio per node. Unlike GPSR, TEF has the highest average retransmission ratio per node among three face-routing techniques because it usually selects the farthest candidate nodes with the low data transmission success ratio due to the farthest transmission distance. The proposed scheme has the better average retransmission ratio per node compared with TEF. Since the proposed scheme selects the farther candidate nodes with the higher data transmission success ratio among them, it can reduce the data retransmission to the candidate nodes of face-routing. However, as shown in this Figure 12, since the proposed scheme tries to transmit the data message to the farther neighbor nodes in one-hop range, this scheme has a higher average retransmission ratio per node when it is compared with GPSR. To sum up, GPSR has the lowest average retransmission ratio per node compared with two face-routing techniques, TEF has the highest average retransmission ratio per node, and the proposed scheme is located in the middle position between two related face-routing techniques.

## 5. Conclusions

In geographic routing, face-routing is one of the recovery schemes to solve the failure of data transmission. Although face-routing research can solve the failure of geographic routing, due to rule that only follows the planar graph of face-routing, it leads to unnecessary frequent data transmission between the source node and the destination node in one-hop range and, therefore, this case decreases the energy efficiency of nodes. To improve energy efficiency, recent face-routing research can reduce overall data transmission of face-routing by transmitting the farthest-neighbor nodes in one-hop range. However, since transmission reliability decreases with transmission distance, these techniques cause lots of data retransmission due to their farthest-node selection in one-hop range. To solve this problem, we propose a novel face-routing scheme that considers the balance between transmission distance and transmission reliability by using two viewpoints. The first viewpoint targets how to increase the transmission distance in one-hop range. The proposed scheme searches the farther candidate nodes of face-routing in one-hop range by using the advantage of the farthest-node selection research in face-routing to improve energy efficiency of nodes. After that, the second viewpoint points out how to increase transmission success ratio of delivery. The proposed scheme transmits data packets to the candidate nodes with a good-condition link among the selected candidate nodes in the first viewpoint to improve the transmission reliability. By exploiting two viewpoints, the proposed scheme can transmit data packets to appropriate neighbor nodes in one-hop range in terms of energy efficiency and transmission reliability. By using two viewpoints, the proposed scheme can get an appropriate balance between energy efficiency and data transmission reliability. In the simulation, the proposed method shows better performance in terms of energy efficiency than existing face-routing research, and it is also better than recent research in face-routing in terms of reliability and retransmission.

## Figures and Tables

**Table 1:** The Components of the Simulation Environment.

Simulation Component	Values
The network simulator	MATLAB [33]
Terrain	(80 m, 80 m)
The number of the nodes	500 nodes (from 200 to 500)
End-to-end distance	70 m (from 60 to 74)
Node deployment	Randomly placement
MAC protocol	802.15.4 MAC
Transmission range	30 m (from 30 m to 40 m)
Simulation repetition	200,000 times
Energy consumption (TX)	57.42 mW [34,35]
Energy consumption (RX)	62.04 mW [34,35]
Comparison targets	GPSR [6], TEF [18], Proposed scheme

## Data Availability

Not applicable.
